# 2359. Relative effectiveness of mRNA-1273, BNT162b2, and Ad26.COV2.S vaccines in adults at higher risk for severe COVID-19 outcomes

**DOI:** 10.1093/ofid/ofad500.1980

**Published:** 2023-11-27

**Authors:** Van Hung Nguyen, Catherine Boileau, Alina Bogdanov, Ni Zeng, Mac Bonafede, Thierry Ducruet, Andrew M Rosen, David Martin, Daina Esposito, Nicolas Van de Velde, Hagit Kopel, James Mansi

**Affiliations:** VHN Consulting Inc., Montreal, Quebec, Canada; VHN Consulting Inc., Montreal, Quebec, Canada; Veradigm, Chicago, Illinois; Veradigm, Chicago, Illinois; Veradigm, Chicago, Illinois; VHN Consulting Inc., Montreal, Quebec, Canada; Moderna, Inc., Cambridge, Massachusetts; Moderna, Inc., Cambridge, Massachusetts; Moderna, Inc., Cambridge, Massachusetts; Moderna, Inc., Cambridge, Massachusetts; Moderna, Inc., Cambridge, Massachusetts; Moderna

## Abstract

**Background:**

Older age ( > 65 years) and underlying chronic medical conditions are risk factors associated with severe COVID-19 outcomes. In this study, we evaluated the relative vaccine effectiveness (rVE) of a primary series of mRNA-1273 (2 doses) versus BNT162b2 (2 doses) or Ad26.COV2.S (1 dose) and rVE of monovalent mRNA boosters - against medically attended (outpatient and hospitalizations cases), outpatient, and hospitalization cases due to COVID-19 in adults ≥ 18 years with at least one underlying medical condition and by age groups.

**Methods:**

Data from a U.S. electronic health records system linked with medical claims data were used to evaluate rVE in patients with at least one underlying medical condition (such as immunosuppression, diabetes, chronic lung diseases, cardiovascular diseases, and renal diseases). Part 1 of the study evaluated the rVE of the primary series (February to October 2021), and Part 2 evaluated the rVE of a single monovalent mRNA booster dose (October 2021 to January 2022). Individuals were matched by sex, geographic region, age group, and race.

**Results:**

In Part 1, mRNA-1273 prevented more medically-attended COVID-19 cases than BNT162b2 and Ad26.COV2.S, with rVEs of 24% (95% confidence interval [CI]: 22–25%) and 51% (49–52%), respectively. Similarly, mRNA-1273 also prevented more outpatient and hospitalized COVID-19 cases than the two comparator vaccines (**Table 1**). Findings were consistent across age groups. In Part 2, rVE of mRNA-1273 vs. BNT162b2 against medically-attended COVID-19 cases was 14% (95% CI: 9-19%). Importantly, following a booster dose, mRNA-1273 prevented more hospitalizations than BNT162b2 with an rVE of 22% (95% CI: 3–37%) in the overall study population and increased with age, with estimates of 32% (13–47%) and 46% (19–65%) in adults ≥ 50 years and ≥ 65 years, respectively.

**Figure:**
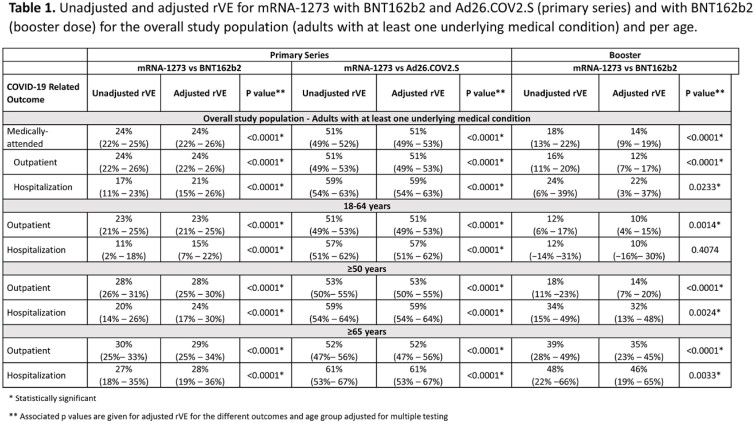

**Conclusion:**

In this study, a primary series of mRNA-1273 was more effective than BNT162b2 or Ad26.COV2.S in preventing medically-attended COVID-19 in individuals at higher risk for severe COVID-19 outcomes. Moreover, a booster dose of mRNA-1273 was more effective compared with BNT162b2, with additional significant benefits against COVID-19-related hospitalizations in older adults.

**Disclosures:**

**Van Hung Nguyen, MPH**, Moderna, Inc.: Advisor/Consultant|VHN Consulting Inc.: Salary **Catherine Boileau, PhD**, Moderna, Inc.: Advisor/Consultant|VHN Consulting Inc.: Salary **Alina Bogdanov, MA**, Moderna, Inc.: Advisor/Consultant|Veradigm: Salary **Ni Zeng, PhD**, Moderna, Inc.: Advisor/Consultant|Veradigm: Salary **Mac Bonafede, PhD**, Moderna, Inc.: Advisor/Consultant|Veradigm: Salary **Thierry Ducruet, MSc**, Moderna, Inc.: Advisor/Consultant|VHN Consulting Inc.: Salary **Andrew M. Rosen, PhD**, Moderna, Inc.: Salary|Moderna, Inc.: Stocks/Bonds **David Martin, MD, MPH**, Moderna, Inc.: Salary|Moderna, Inc.: Stocks/Bonds **Daina Esposito, PhD, MPH**, Moderna, Inc.: Salary|Moderna, Inc.: Stocks/Bonds **Nicolas Van de Velde, PhD**, Moderna, Inc.: Salary|Moderna, Inc.: Stocks/Bonds **Hagit Kopel, PhD**, Moderna, Inc.: Salary|Moderna, Inc.: Stocks/Bonds **James Mansi**, Moderna, Inc.: Salary|Moderna, Inc.: Stocks/Bonds

